# Non-homologous End Joining-Mediated Insertional Mutagenesis Reveals a Novel Target for Enhancing Fatty Alcohols Production in *Yarrowia lipolytica*

**DOI:** 10.3389/fmicb.2022.898884

**Published:** 2022-04-25

**Authors:** Mengxu Li, Jinlai Zhang, Qiuyan Bai, Lixia Fang, Hao Song, Yingxiu Cao

**Affiliations:** ^1^Frontier Science Center for Synthetic Biology and Key Laboratory of Systems Bioengineering (Ministry of Education), School of Chemical Engineering and Technology, Tianjin University, Tianjin, China; ^2^Key Laboratory of Systems Bioengineering (Ministry of Education), Tianjin University, Tianjin, China

**Keywords:** new target identification, non-homologous end joining-mediated integration, *Yarrowia lipolytica*, fatty alcohols, RNA-Seq

## Abstract

Non-homologous end joining (NHEJ)-mediated integration is effective in generating random mutagenesis to identify beneficial gene targets in the whole genome, which can significantly promote the performance of the strains. Here, a novel target leading to higher protein synthesis was identified by NHEJ-mediated integration that seriously improved fatty alcohols biosynthesis in *Yarrowia lipolytica*. One batch of strains transformed with fatty acyl-CoA reductase gene (*FAR*) showed significant differences (up to 70.53-fold) in fatty alcohol production. Whole-genome sequencing of the high-yield strain demonstrated that a new target YALI0_A00913g (“A1 gene”) was disrupted by NHEJ-mediated integration of partial carrier DNA, and reverse engineering of the A1 gene disruption (YlΔA1-FAR) recovered the fatty alcohol overproduction phenotype. Transcriptome analysis of YlΔA1-FAR strain revealed A1 disruption led to strengthened protein synthesis process that was confirmed by *sfGFP* gene expression, which may account for enhanced cell viability and improved biosynthesis of fatty alcohols. This study identified a novel target that facilitated synthesis capacity and provided new insights into unlocking biosynthetic potential for future genetic engineering in *Y. lipolytica*.

## Introduction

The metabolic reactions and regulatory mechanisms that exist in organisms are not performed in isolation, thereby microbial biosynthesis of a desired product is constantly in the control of a complex intracellular network (Herrgard et al., 2006; Chen et al., 2018). In addition to the synthetic pathway, an abundance of beneficial gene targets in the genome can promote the performance of strains, such as production yield (Wang et al., 2018), toxicity tolerance (Hu et al., 2021; Li et al., 2021) or cell viability (Shiomi et al., 2013; Jiang et al., 2015). However, identification of potential targets of unknown function by rational engineering remains difficult because of our limited understanding of the underlying linkages among the metabolic processes (Ajjawi et al., 2017; Fang et al., 2021). In contrast, random mutagenesis succeeds in inducing arbitrary mutations with random distribution to produce genotypic diversity, which is an efficient approach to identify new targets and allow further unravel the inner machinery of metabolism by mapping the genotypes and phenotypes (van Opijnen et al., 2009).

Non-homologous end joining (NHEJ) is a typical pathway to repair genomic DNA double-strand breaks (DSBs; Lieber, 2010; Ranjha et al., 2018). NHEJ is active throughout the cell cycle and is considered the predominant DSB repair pathway (Bouwman and Crosetto, 2018). In human cells, NHEJ appears to repair nearly all DSBs outside of S and G2 cell cycle phases and even about 80% of DSBs within S and G2 (Beucher et al., 2009). NHEJ-mediated integration can efficiently generate random mutagenesis across the genome in *Yarrowia lipolytica*. Firstly, NHEJ repairs DSBs is recognized as error-prone, which tends to result in gene mutations and is rarely restored to their original DNA sequence (Burma et al., 2006; Shrivastav et al., 2008; Pannunzio et al., 2018). Secondly, NHEJ-mediated integration confers the randomness and genome-scale unbiased coverage of gene mutations in *Y. lipolytica*. Liu et al. revealed that 9.15 × 10^5^ NHEJ-mediated independent integration events were relatively uniformly distributed across the genome among approximately one million colonies (Liu et al., 2022). Finally, NHEJ-mediated integration is time-saving and convenient to generate gene mutations. Compared with other mutagenesis approaches, NHEJ-mediated integration requires only linear DNA amplification without the assistance of heterologous transposons (Kumar, 2016) or a CRISPR system (Schwartz et al., 2019) and therefore the mutations are generated easily and rapidly. Up to 65 copy numbers were achieved within 96 h by Bai et al. through optimized NHEJ-mediated genome integration technology (Bai et al., 2021). Based on these advantages, NHEJ-mediated integration was utilized to screen causal mutations related to interested phenotypes. For example, 7 new targets for improving β-carotene biosynthesis or acetic acid tolerance were identified by NHEJ-mediated insertional mutagenesis in *Y. lipolytica* (Liu et al., 2022), which contributed to superior cell factory construction.

*Yarrowia lipolytica* displays a strong preference for repairing DSBs by NHEJ other than homologous recombination (HR; Kretzschmar et al., 2013; Verbeke et al., 2013). Besides the complete foreign sequences, even the fractional fragments or vector can also be spontaneously inserted into the genome, resulting in various genotypes in one batch of gene transformation (Liu et al., 2019; Longmuir et al., 2019). However, the potentially beneficial gene targets might be missed, if the researchers only select strains with phenotypes of interest without further analysis of random insertion sites (Ye et al., 2012). For example, if the whole-genome sequencing had not been performed on a high-yield eicosapentaenoic acid producing strain generated by NHEJ-mediated random insertion of pathway genes, mutation of *PEX10* gene might have been overlooked (Xue et al., 2013), which was proved to be highly effective and was widely engineered to enhance lipids and terpenoids production in later works (Blazeck et al., 2014; Jin et al., 2019; Wei et al., 2021). Therefore, the incidental phenotypic differences should be valued and further investigated, which might contribute to the identification of potential targets and greatly expand the understanding of biological knowledge.

In this study, we spotted unexpected strains with fatty alcohols overproduction in the same batch after transforming the fatty acyl-CoA reductase (*FAR*) gene. Then, the whole-genome sequencing of the high-yield strains showed that the YALI0_A00913g (“A1 gene”) was disrupted due to the partial carrier DNA sequence was inserted into the open reading frame (ORF) by NHEJ-mediated integration. Reverse engineering of the A1 gene disruption (YlΔA1-FAR) recovered the high-yielding fatty alcohols phenotype. Transcriptome analysis of YlΔA1-FAR strain revealed that disruption of A1 gene resulted in a significant enhancement of protein synthesis, which was confirmed by characterization with green fluorescence intensity (*sfGFP* gene). Therefore, the disruption of new target A1 boosted the cell viability and accounted for the increased fatty alcohol production. Target identification by NHEJ-mediated integration facilitated the unlocking of biosynthetic potential and effectively increased the productivity of *Y. lipolytica*.

## Materials and Methods

### Strains, Medium and Culture Conditions

*Yarrowia lipolytica* strain ATCC 201249 (MATA, ura3-302, leu2-270, lys8-11, PEX17-HA) was selected as the background strain for all constructs (Gao et al., 2016), and the initial strain ATCC 201249 was provided by Professor Ying-Jin Yuan (Tianjin University, China). All derivatives constructed in this study are listed in Supplementary Table 1. The above strains of *Y. lipolytica* were cultured in yeast culture medium at 28°C and 250 rpm shaking speed.

TransT1 *E. coli* was used for plasmid construction and propagation, which was grown in Luria-Bertani broth (LB) at 37°C with constant shaking at 220 rpm. When necessary, the selective antibiotic was added (100 mg/l ampicillin or 50 mg/l kanamycin). Agar (15 g/l) was added for LB solid plate preparation. Yeast extract-peptone-dextrose (YPD) medium, consisting of 20 g/l glucose, 20 g/l peptone, and 10 g/l yeast extract, was used for transformation, activation and preculture. Agar (20 g/l) was added for YPD solid plate preparation. The rich YPD medium containing 50 g/l glucose, 20 g/l peptone, and 10 g/l yeast extract was used for fermentation of *Y. lipolytica* strains. The SC medium, used for screening *Y. lipolytica* transformants, contained 20 g/l glucose, 6.7 g/l yeast nitrogen base without amino acids, and 2 g/l complete supplement mixture (CSM) lacking uracil (SC-Ura) or leucine (SC-Leu), supplemented with uracil or leucine depending on the selection marker requirements.

For fatty alcohols flask fermentation, freshly streaked single colonies of strains were first cultivated in a 25-mL polypropylene tube with 5 ml YPD medium, and cultivate overnight at 28°C with a shaking speed of 250 rpm. After preculturing, the seed cultures were inoculated into 50 ml of fresh YPD medium with an initial OD600 of 0.2 and fermented in a 250-mL shake flask for 72 h or extend appropriately.

### Construction of Plasmids and Strains

Plasmids used in this work are listed in Supplementary Table 2. Fatty acyl-CoA reductase gene from *Marinobacter aquaeolei* VT8 (i.e., *MaFAR*) and *sfGFP* were codon optimized and synthesized by GenScript (Nanjing, China) and listed in Supplementary Data 1. The IntX HUM were amplified from genomic DNA of *Y. lipolytica* and constructed the integrated plasmids pIntA, pIntB, pIntC, pIntD and pIntE through seamless cloning. And the expressed gene were amplified with the proper sticky ends, cleaved with DNA endonuclease and integrated into the corresponding integration plasmids by T4 DNA ligase. All the primers used to construct the expression cassettes and the plasmids were synthesized by Genewiz Inc. (China) and are listed in Supplementary Table 3. The genotypes of all strains were confirmed by colony polymerase chain reaction of KOD FX DNA polymerase (Toyobo Co., Ltd.; Shanghai, China).

For disrupting target genes in *Y. lipolytica*, the CRISPR system was used as previously described (Schwartz et al., 2016; Zhang et al., 2018). The backbone plasmid used for constructing CRISPR-Cas9 plasmids for gene knockout in *Y. lipolytica* was pMCS-Cen1. The synthesized gRNA was incorporated into pMCS-URA *via* one-step Golden Gate assembly. The corresponding plasmids were digested with restriction enzyme BamHI and HindIII, and ligated with the segment of Cas9 to form the final plasmids. Strains with Ura-marker plasmid were inoculated into YPD liquid medium and cultured at 28°C for 3–4 days, then they were screened on YPD solid medium containing 1.2 mg/ml 5-fluoroorotic acid after dilution for the loss of Ura-marker plasmid.

### Transformation

*Yarrowia lipolytica* transformation with integrative fragments or recombinant plasmids was performed with Zymogen Frozen EZ Yeast Transformation Kit II (Zymo Research Corporation). For CRISPR plasmid transformations, the cells were transformed with plasmid and cultivated in SC-Ura liquid medium for 4 d. Then the cells were plated onto SC-Ura plates for 2 d and confirmed by sequencing. For integrative fragment transformations, approximately 2 μg of linearized DNA was used in the transformation reaction and then the cells were harvested by centrifugation at 5,000 rpm for 2 min and plated on SC agar plates without the auxotrophic compound supplemented by the fragments. Selection plates were incubated at 28°C for 2–3 days.

### Extraction and Analysis of Fatty Alcohols

Extraction of fatty alcohols was similar with the previously described method (Xu et al., 2016). Briefly, cells from 0.5 ml of culture were blended with 1-Nonadecanol acid (the final concentration is about 50~500 mg/l, adjusted according to the actual output) as internal standard (IS) and extracted with 0.5 ml of ethyl acetate. The ethyl acetate extract was vortexed at a speed of 1,200 r/min for 20 min, and then centrifuged at 15000 rpm for 25 min. The supernatant organic phase was filtered through a 0.22 μm filter membrane for fatty alcohols assay using gas chromatography (GC). The extracted fatty alcohol was then determined using a Thermo Scientific TRACE 1300 GC equipped with a TG-5MS column (30 m × 0.32 mm × 0.25 μm; Thermo Scientific) and a Flame Ionization Detector (FID) operating under constant flow rate of the carrier gas (nitrogen) at 1 ml min^−1^. The injector temperature was 260°C; it was operated on constant pressure mode at 91 kPa. The column temperature was held at 70°C for 2 min and then increased to 290°C with the rate of 8°C/min, holding for 6 min. Individual fatty alcohol species were qualified by authentic homologous standards and quantified by comparing the peak areas with that of the internal standard using the Chromeleon 7.1 software.

### Fluorescence Assay

The *Y. lipolytica* strains transformed with *sfGFP* gene were activated in Sc-Leu medium for 24 h, and then the seed cultures were inoculated into 25-mL polypropylene tube containing 5 ml fresh SC-Leu medium with an initial OD600 of 0.2. The strains were grown at 250 rpm for 48 h at 28°C. 500 ul suspensions of each polypropylene tubes were centrifuged at 5000 rpm for 2 min to remove the supernatant and the cells were washed and resuspended with the phosphate-buffered saline (PBS) buffer. Fluorescence intensity (excitation: 488 nm and emission: 530 nm) was measured using a 96-well polystyrene plates (black plate, clear bottom with lid; Corning Incorporated 3,603, United States) after dilution into the linear range of the detector by a multimode microplate reader (Tecan Infinite 200 PRO, Austria). The fluorescence intensity was normalized to cell density (OD600), which was measured using the same microplate reader. The images of *sfGFP* fluorescence were observed by fluorescence microscope (Olympus CX41, Japan). Experiments were performed in three biological replicates.

### Whole-Genome Sequencing

Whole-genome sequencing of *Y. lipolytica* strains was performed with an Illumina Hiseq2000 by the Genomic Sequencing and Analysis Facility at the Beijing Genome Institute (BGI, China) using paired-End sequencing. Genomic DNA of each individual is extracted by CTAB method (Murray and Thompson, 1980), and then fragmented randomly. After electrophoresis, DNA fragments of desired length are gel purified. Adapter ligation and DNA cluster preparation are performed and subjected to sequencing. Step one, the sequencing reads are aligned onto the reference CLIB122 genome sequence using SOAPaligner,^1^ obtain the single reads on the alignment, and perform pre-processing on this part of the reads. The sequencing depth and coverage compared to the reference genome are calculated based on the alignment. Step two, align the sequencing reads with the insert sequence as a reference sequence, and obtain the single reads aligning with the insert sequence. Extract the intersection of the two steps, count the distribution of reads in the genome, and determine the insertion site. SOAPsnp,^2^ SOAPindel and SOAPsv were used to analyze the data.

### RNA-Seq Transcriptomic Analysis

*Yarrowia lipolytica* cells cultured at 24 h on the initial logarithmic phase were collected for RNA isolation. Cells were collected by centrifugation at 5000 rpm for 2 min, washed with PBS three times and then quick-frozen by liquid nitrogen and stored at − 80°C tentatively. The RNA sample preparation, library construction and sequencing were performed at the Beijing Genome Institute (BGI, China). RNA was isolated from the cell pellet using TRIzol^™^ Reagent (Invitrogen, United States), and the quality was determined using Bioanalyzer 2,100 (Agilent, United States). The RNA was sheared and reverse transcribed using random primers to obtain cDNA which was used for library construction. Then, the library quality was determined using a Bioanalyzer 2100 (Agilent) analyzer, and the libraries were sequenced on the BGISEQ-500 (BGI, China) sequencing platform. All raw sequencing reads were filtered using BGI’s own filtering software SOAPnuke to remove reads containing low quality, splice contamination and unknown base N content greater than 5%. The filtered “Clean Reads” were saved in FASTQ format for data analysis to ensure reliable results. Gene expression levels were calculated for each sample using the RSEM (Li and Dewey, 2011; Langmead and Salzberg, 2012) software package. NOI Seq (Anders and Huber, 2010; Tarazona et al., 2011) method for detecting differential gene expression between groups. Finally, in-depth data analysis was performed on the BGI data analysis platform.^3^

### Determination of ATP Level

Cellular ATP levels were determined using an Enhanced ATP Assay Kit (Beyotime S0027, Nanjing, China). Equal numbers of *Y. lipolytica* cells cultured at 24 h on the initial logarithmic phase were collected and lysed by an ultrasonic cell-crushing device was used for cell disruption (COLE-PARMER INSTRUMENTS CP505, United States), which were centrifuged for 5 min at 4°C and 12,000 g, and the supernatant was collected. Subsequently, 100 ul of ATP detection solution was added to a 96-well plate (Corning Incorporated 3,603, United States) and then 20 ul of the supernatant was transferred into each well. Chemiluminescence was monitored using a multimode microplate reader (Tecan Infinite 200 PRO, Austria).

## Results

### Incidental Obtaining of Strains With High Fatty Alcohols Producing

**Figure 1:**
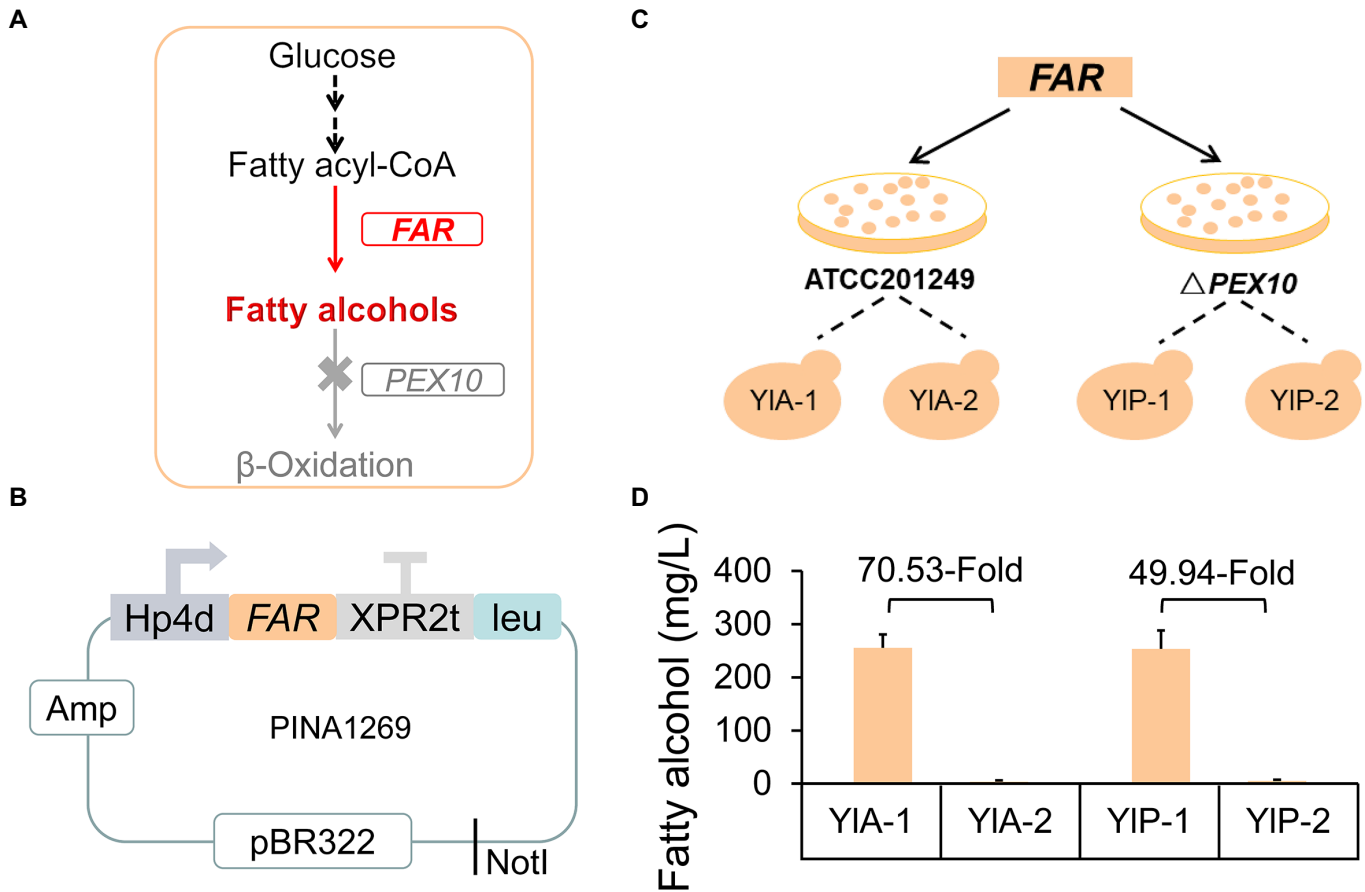
Strains with different fatty alcohols yields were found in the same batch of transformed of *FAR* gene. **(A)** Schematic diagram of the fatty alcohol biosynthesis pathway engineering strategies in *Yarrowia lipolytica*. *FAR*, fatty acyl-CoA reductase gene from *Marinobacter aquaeolei* VT8; *PEX10*, the peroxisome synthesis gene. Fatty acyl-CoA reductase converts fatty acyl-CoA to fatty alcohols. The gene *PEX10* was deleted to prevent the degradation of fatty alcohols. **(B)** Schematic diagram of the plasmid pINA1269-*FAR* used to transform *Y. lipolytica*. The *FAR* gene was driven by the Hp4d promoter and the plasmid was linearized by NotI and transformed into different basic strains and two transformants were randomly selected from each plate for fermentation **(C)**. **(C)** Schematic diagram of establishing exogenous synthesis pathway of fatty alcohols. **(D)** Fatty alcohols production of strains from the same batch of transformants. All strains were cultured in rich YPD medium. Samples were taken at 72 h. Data are presented as mean ± SD of three biological replicates.

Fatty alcohols have gained huge market and applied value due to their broad applications in the cosmetics, detergent, surfactant, and lubricant industries (Pfleger et al., 2015; Fillet and Adrio, 2016). It has been previously proven that fatty acyl-CoA reductase from *M. aquaeolei* VT8 (encoded by the *MaFAR* gene) was the most effective enzyme to directly catalyze the reduction of fatty acyl-CoA to fatty alcohols (Figure 1A; Wahlen et al., 2009; Willis et al., 2011; Wang et al., 2016b). To establish an exogenous synthesis pathway of fatty alcohol in *Y. lipolytica*, the *MaFAR* gene was expressed by pINA1269 vector in strains ATCC 201249, and Y10301 (Δ*PEX10*; Zhang et al., 2019), generating strains YlA and YlP (Figures 1B,C). pINA1269 is a pBR322-based mono-copy integrative vector carrying the LEU2 gene, which is supposed to integrate into the PBR docking platform of the genome through homologous recombination (Xuan et al., 1990; Madzak et al., 2000; Nicaud et al., 2002). We picked a series of strains randomly on each plate for fermentation; however, the results were amazing. Strains with unexpected yields were found incidentally and the yield of other strains was at the general level. As shown in Figure 1D, strains from the same plate have significantly different yields and we selected one of the low-yielding strains as the control. Strain YlA-1 produced 255.3 mg/l of fatty alcohols, which was 70.53-fold higher than strain YlA-2 (3.62 mg/l), both two strains based on ATCC 201249. Similarly, YlP-1 produced 49.94-fold fatty alcohols than YlP-2. In theory, the strains obtained from the same transformation batch should not have significant differences in fatty alcohols production. But NHEJ is the main pathway for repairing DNA double strand breaks (DSB) of genome in *Y. lipolytica* (Richard et al., 2005; Verbeke et al., 2013), which may produce unexpected NHEJ-mediated insertions accompanied by integration of the pINA1269-FAR plasmid to generate new genotypes (Gao et al., 2014; Cui et al., 2019; Sudfeld et al., 2021).

### Whole-Genome Sequencing Suggested Gene Disruption by NHEJ-Mediated Integration

**Figure 2:**
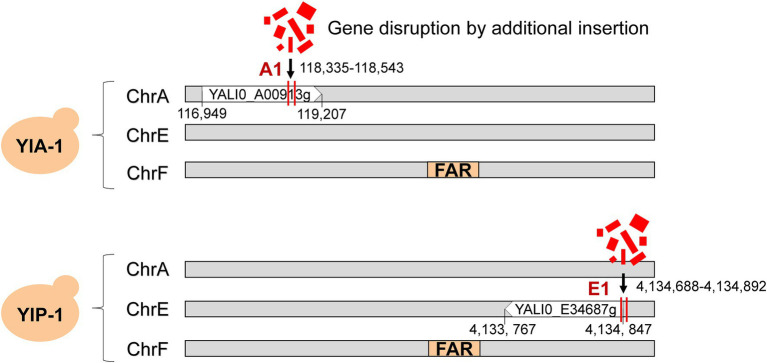
Whole-genome sequencing determined gene disruption in fatty alcohol high-yielding strains. YlA and YlP strains were applied to the whole-genome sequencing. The *FAR* gene marked in orange was integrated into the chromosome F as expected. The gene disruption caused by the integration of broken vector fragments into chromosomes A and E was marked with red vertical line, named A1 and E1 sites, respectively. The A1 site was located in the 118,335–118,543 region of chromosomes A, and the E1 site was in the 4,134,688–4,134,892 region of chromosomes E.

To further investigate the potential cause of improved fatty alcohols production, we performed whole-genome sequencing of the YlA-1,2 and YlP-1,2 to confirm the genotypes (Liu et al., 2015a,b; Li and Alper, 2016). As shown in Figure 2, the *FAR* gene was integrated into the pBR322 locus on chromosome F as expected by comparison with the draft genome sequence of the wild-type control strain, ATCC 201249. However, additional changes of gene disruption were indeed discovered in genome of high-yield strains, which were caused by the integration of a small proportion of vector into chromosomes A and E, named A1 and E1 sites, respectively. The specific locations of disruptions are shown in Figure 2; the A1 site was located in the 118,335–118,543 region of chromosomes A, which was in the open reading frame in YALI0_A00913g gene (“A1 gene”). Similarly, the E1 site was in the 4,134,688–4,134,892 region of chromosomes E in YALI0_E34687g gene (“E1 gene”). However, these disruptions were not available in low-yield strains of YlA-2 and YlP-2 (Supplementary Figure 1; Supplementary Table 4), and we hypothesized that these additional changes led to high yield of fatty alcohols.

### Reverse-Engineered Disruption Validation of New Target YALI0_A00913g (A1 Gene)

**Figure 3:**
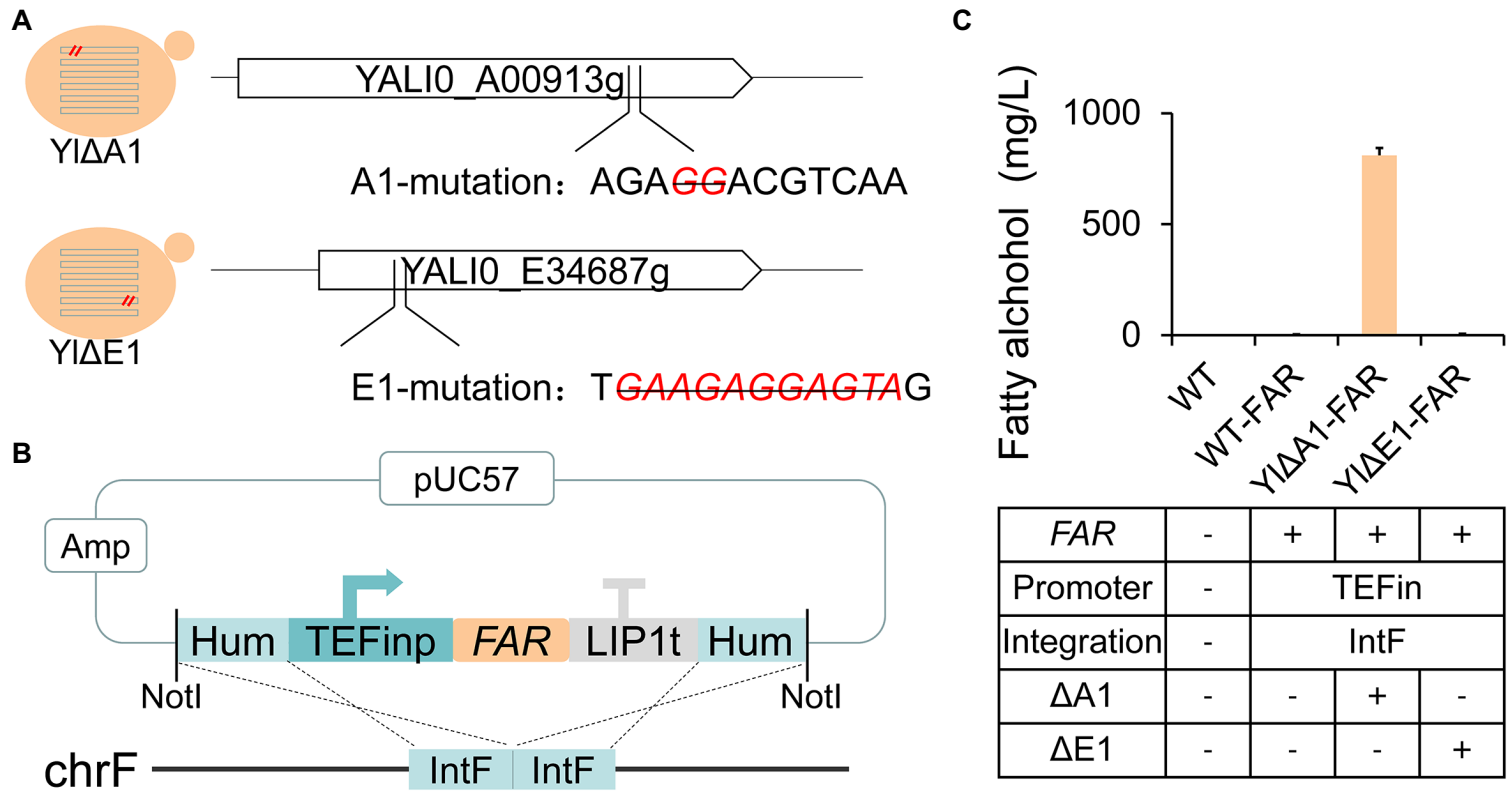
Reverse engineering of the candidate targets derived from WGS to identify beneficial gene for enhanced fatty alcohol production. **(A)** CRISPR/Cas-mediated knockout of candidate gene targets in wild-type *Y. lipolytica* strain ATCC 201249. The deletion of bases at A1 and E1 sites in ATCC 201249 genome were marked with red and strikethrough. The knockout location is contained within the location of the broken vector fragment insertion. **(B)** Schematic diagram of the plasmid pIntF-*FAR* used to transform *Y. lipolytica*. The *FAR* gene is driven by the TEFin promoter and flanked by 2 homology arms of IntF location. The plasmid was linearized by NotI and transformed into different basic strains and two transformants were randomly selected from each plate for fermentation. **(C)** The effect of A1 and E1 sites disruption on fatty alcohols production. All strains were cultured in rich YPD medium. Values are the mean of three biological replicates ± standard deviation (*n* = 3) after 72 h.

We further conducted reverse engineering on these targets to validate whether the disruption of unknown genes A1 and E1 can enhance the fatty alcohols production. As shown in Figure 3A, the A1 and E1 genes were deleted 2 bp (GG) and 11 bp (GAAGAGGAGTA), respectively, on the basis of the blank strain ATCC 201249 through the CRISPR/Cas system (Gao et al., 2016; Schwartz et al., 2016), obtaining the mutant strains YlΔA1 and YlΔE1. The position we selected to knock out was consistent with the site of the broken vector fragments insertion in the previous whole-genome sequencing. To reduce the occurrence of the incidental insertion by NHEJ, we selected a relatively stable pIntF vector contained 500 bp homologous to IntF (YALI0F3161413 to YALI0F3162449) flanking a gene expression cassette (Matthaus et al., 2014; Holkenbrink et al., 2018; Figure 3B). The *FAR* gene was expressed under the control of TEFin promoter by pIntF vector and integrated into the IntF site in strains ATCC 201249, YlΔA1 and YlΔE1, respectively. Three strains were generated, named WT-FAR, YlΔA1-FAR and YlΔE1-FAR. Fatty alcohols production for 72 h of fermentation was shown in Figure 3C, the yield of strain WT-FAR without knockout target was only 2.47 mg/l and the strain YlΔE1-FAR that knocked out the E1 target produced 4.50 mg/l of fatty alcohols. Previous studies have shown that the yield of *Y. lipolytica* strains expressing the *MaFAR* gene is only 1.5–80 mg/l (Xu et al., 2016; Wang et al., 2016a; Zhang et al., 2019), so the low-yield strains are conventional. While the yield of strain YlΔA1-FAR with knockout A1 target is as high as 810.39 mg/l (Fermentation for 90 h up to 851.8 mg/l, Supplementary Figure 2), which was even higher than the yield of previous strains optimized for metabolism (Wang et al., 2016a; Xu et al., 2016; Zhou et al., 2016), which is unconventional. It was confirmed that the disruption of A1 gene can significantly increase the yield of fatty alcohols, but the disruption of E1 gene did not reach the expected yield. Since the high fatty alcohol producing strain YlP-1 was based on Yl0301 (Δ*PEX10*), but we deleted the target gene E1 in ATCC 201249 instead of Y10301. This may have resulted in the YlΔE1-FAR not reaching the expected yield.

### Transcriptome Analysis Revealed the Increased Protein Synthesis Owing to A1 Disruption

**Figure 4:**
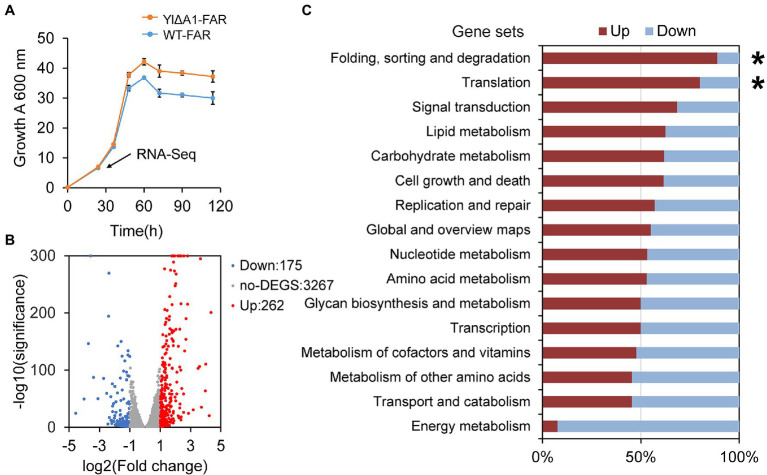
Transcriptome analysis of YlΔA1-FAR and WT-FAR. **(A)** Growth rate of YlΔA1-FAR strain with high fatty alcohols production compared with WT-FAR under rich medium conditions. All cultivations were performed in rich YPD medium for 108 h. Transcriptome analysis (RNA-Seq) was performed on the samples at 24 h (pointed by arrow). Results are presented as mean ± s. d. of three biological replicates. **(B)** Fold changes (log2) of genes differentially expressed (adjusted *p* < 0.05, |Fold change| > 1) in YlΔA1-FAR cells relative to WT-FAR cells. Genes at the positive side of the x axis are upregulated (highlighted in red), and genes at the negative side are downregulated (highlighted in blue). **(C)** KEGG pathways enrichment analysis. Gene sets were defined by KEGG pathways, which showed the percentage of genes that were either up- (red) or down-regulated (blue).

The hypothetical gene A1 (YALI0_A00913g) was weakly similar (31.4%) to the *YND1* gene of *Saccharomyces cerevisiae* (D’Alessio et al., 2005; Maoz et al., 2005; Onuma et al., 2006; Mittelman et al., 2010) after alignment by Protein BLAST (Supplementary Figure 3). The *YND1* gene encodes Golgi apyrase that is not directly related to the fatty alcohol biosynthesis pathway. In order to reveal the possible mechanism of high yield caused by the disruption of A1 gene, RNA-Seq was performed to analyze the transcriptome differences between YlΔA1-FAR and WT-FAR. As shown in Figure 4A, cell samples were taken at 24 h which was the initial logarithmic phase. Transcriptome analysis results showed that the disruption of A1 gene and excess accumulation of fatty alcohols resulted in large transcriptional changes. A total of 437 DEGs were identified in YlΔA1-FAR compared with the control, with 262 and 175 genes being upregulated and downregulated, respectively (Figure 4B). The DEGs were subjected to KEGG enrichment analysis, which were enriched in 16 pathways. Among them, the folding, sorting and degradation and translation noted by asterisks (*) are the top two gene sets with the highest proportions of upregulated genes, reaching 88.9 and 80.0%, respectively (Figure 4C). The two gene sets were obviously related to protein synthesis and processing, and the genes with significant differences in transcription level are shown in Table 1.

**Table 1:** The differentially expressed genes in the folding, sorting and degradation and translation gene sets.

Functional group and gene	log2-Fold	Description
**Translation**		
RNA transport		
YALI0_C17567g	2.56	NUP98, ADAR2; nuclear pore complex protein Nup98-Nup96 (Ho et al., 2000; Krull et al., 2004)
YALI0_D20658g	1.30	UPF2, RENT2; regulator of nonsense transcripts 2 (Chamieh et al., 2008)
Ribosome		
YALI0_B13200g	2.66	EIF3B; translation initiation factor 3 subunit B (Phan et al., 2001)
YALI0_F25399g	1.62	RP-S15Ae, RPS15A; small subunit ribosomal protein S15Ae (Vladimirov et al., 1996; Ikeda et al., 2017)
YALI0_A02497g	1.44	EIF4G; translation initiation factor 4G (Levy-Strumpf et al., 1997; Gingras et al., 1999)
YALI0_C18975g	1.04	EIF4G; translation initiation factor 4G (Levy-Strumpf et al., 1997; Gingras et al., 1999)
Aminoacyl-tRNA biosynthesis		
YALI0_B13134g	3.18	RARS, argS; arginyl-tRNA synthetase (Mehler and Mitra, 1967; Cavarelli et al., 1998; Delagoutte et al., 2000)
YALI0_C15246g	1.85	LARS, leuS; leucyl-tRNA synthetase (Lincecum et al., 2003)
Folding, sorting and degradation		
Protein translocation		
YALI0_D08635g	1.54	SEC61G, SSS1, secE; protein transport protein SEC61 subunit gamma and related proteins (Falcone et al., 2011)
Protein processing		
YALI0_E35046g	1.74	HSPA1s; heat shock 70 kDa protein 1/2/6/8 (Hunt and Morimoto, 1985; Mayer and Bukau, 2005; Perez-Vargas et al., 2006)
YALI0_E18546g	1.64	HSP20; HSP20 family protein (Sun et al., 2001; Liu et al., 2012)
YALI0_C03465g	1.17	HSP20; HSP20 family protein (Sun et al., 2001; Liu et al., 2012)
Protein degradation		
YALI0_C05709g	−1.26	TRIP12; E3 ubiquitin-protein ligase TRIP12 (Park et al., 2009)
YALI0_A19426g	−1.16	PSMA2; 20S proteasome subunit alpha 2 (Coux et al., 1996; Voges et al., 1999; Smith et al., 2006)

**Figure 5:**
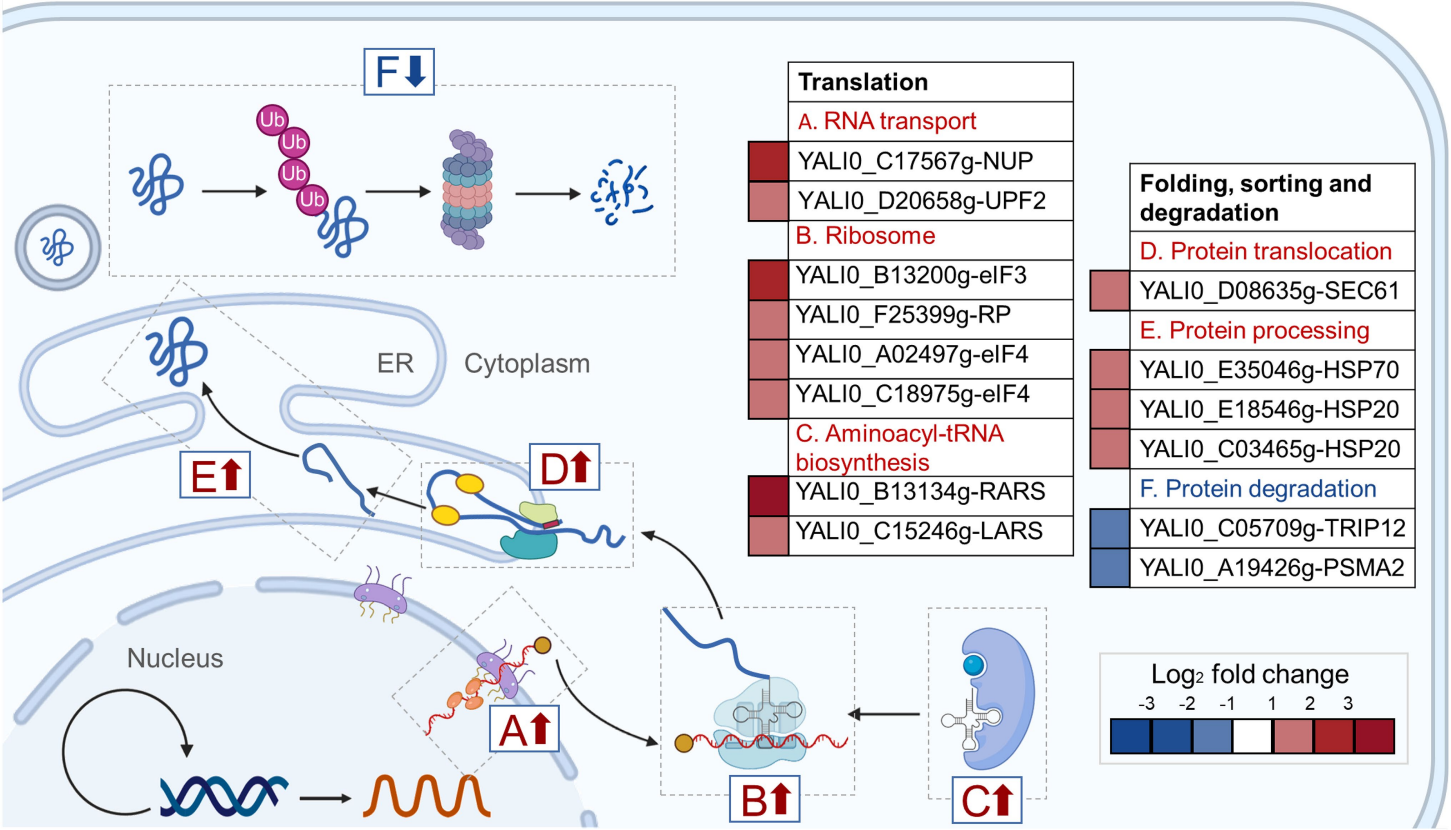
Changes in transcript levels of the protein synthesis process. The **(A)** RNA transport, **(B)** Ribosome and **(C)** Aminoacyl-tRNA synthesis pathway belong to the translation gene set, the **(D)** protein translocation, and **(E)** protein processing and **(F)** protein degradation pathway belong to the folding, sorting and degradation gene set. Among the six pathways, the up- and down-regulated pathways are marked with red and blue arrows, respectively. The boxes indicate fold-change in gene expression of YlΔA1-FAR compared to WT-FAR control. Red boxes indicate upregulated gene expression, and blue boxes indicate downregulation gene expression (*p* < 0.05).

In the translation gene set, 8 genes were significantly upregulated, belonging to RNA transport, ribosome, aminoacyl-tRNA synthesis pathway. First, in the RNA transport pathway, 2 genes YALI0_C17567g and YALI0_D20658g were significantly upregulated by2.56-log2 and 1.30-log2 fold in YlΔA1-FAR strain, respectively. Among them, YALI0_C17567g gene encodes the nuclear pore complex (NPC) protein, which mediates the export of RNAs produced in the nucleus to fulfill their function in protein synthesis (Rout et al., 2000; Rout and Aitchison, 2001; Rodriguez et al., 2004). Therefore, the upregulation of YALI0_C17567g gene can facilitate the transport of RNAs out of the nucleus (Figure 5A). Then, in the ribosome pathway, 4 genes were upregulated in YlΔA1-FAR strain, there (YALI0_B13200g, YALI0_A02497g and YALI0_C18975g) of which are encoding the eukaryotic translation initiation factors (eIFs) family. The initiation of protein synthesis in eukaryotic cells is dependent on multiple eIFs and initiation is the rate-limiting step for translation under most circumstances (Pain and Standart, 2001). Upregulation of eIFs-related genes stimulated the binding of mRNA and methionyl-initiator tRNA (Met-tRNA_i_^Met^) to the ribosome (Gingras et al., 1999; Fromont-Racine et al., 2003; Valasek, 2012), thereby could enhance the translation rates (Figure 5B). Last, two genes YALI0_B13134g and YALI0_C15246g related to the aminoacyl-tRNA biosynthesis pathway were upregulated by 3.18-log2 and 1.85-log2 fold, respectively. They encode aminoacyl-tRNA synthetases (ARS), which catalyze the ligation of amino acids to their cognate tRNAs (Negrutskii and Deutscher, 1991; Cusack, 1997; Park et al., 2008; Ling et al., 2009). The upregulation of related genes facilitated the connection between amino acids and tRNA, thus promoting protein synthesis (Figure 5C).

In the folding, sorting and degradation gene set, genes with significant differences in transcription were mainly distributed in protein translocation, protein processing, and protein degradation pathway. First in the protein translocation pathway, the gene YALI0_D08635g encoding protein transport protein Sec61 subunit gamma was upregulation by 1.54-log2 fold (Falcone et al., 2011). The Sec61 complex mediated protein precursors crossing the endoplasmic reticulum (ER) membrane of eukaryotes, which is a vital, first committed step in the biogenesis of many proteins (Stephenson, 2005; Robson and Collinson, 2006; Rapoport, 2007). Therefore, the upregulation of YALI0_D08635g gene facilitated the precursor proteins to across the ER membrane for subsequent protein processing (Figure 5D). Then, in the protein processing pathway, 3 genes were significantly upregulated, they all belong to the heat shock protein (Hsp) family, which is a kind of molecular chaperone. The Hsp family chaperones prevent protein aggregation and catalyze polypeptide folding in the protein processing (Mayer and Bukau, 2005; Young, 2010; Ghazaei, 2017) and drive post-translational protein translocation to yeast ER (McClellan et al., 1998; Rapoport, 2007; Dudek et al., 2009). The upregulation of related genes promoted the binding of molecular chaperone to hydrophobic patches on proteins (Figure 5E). Finally, in the protein degradation pathway, the YALI0_C05709g gene encoding E3 ubiquitin-protein ligase and the YALI0_A19426g gene encoding 20S proteasome subunit alpha 2 were downregulation by 1.26-log2 and 1.16-log2 fold, respectively. E3s facilitates covalent attachment of ubiquitin to the substrate, then the ubiquitinated protein is recognized and degraded by the 26S proteasome (Johnson et al., 1995; Ciechanover et al., 2000; Park et al., 2009). Thus, downregulation of these genes might reduce protein degradation (Figure 5F).

Above all, transcriptome analysis results suggested that the protein synthesis was significantly enhanced, which potentially contributed to superior cell viability. Our results indicated that the growth of strain YlΔA1-FAR was indeed elevated (Figure 4A) and fatty alcohols production was improved (Figure 3C) compared to the control WT-FAR. As described above, the A1 gene is weakly similar to the *YND1* gene of *S. cerevisiae*, which encodes Golgi apyrase that generally hydrolyzes both ATP and ADP (Supplementary Figure 4A). The transcript levels of the A1 gene before and after disruption are shown in Supplementary Figure 5, which indicated that the disruption did not completely disable the A1 gene but reduced expression level by 50%. The decreased expression of the A1 gene might reduce the consumption of ATP, which may facilitate the maintenance of energy (Choo et al., 2010) and provide energy for protein synthesis. We measured the intracellular ATP levels and the results are shown in Supplementary Figure 4B, the ATP content of YlΔA1-FAR was indeed higher (2.24-fold) than WT-FAR.

### Verification of Improved Protein Expression by *sfGFP* Gene Expression

**Figure 6:**
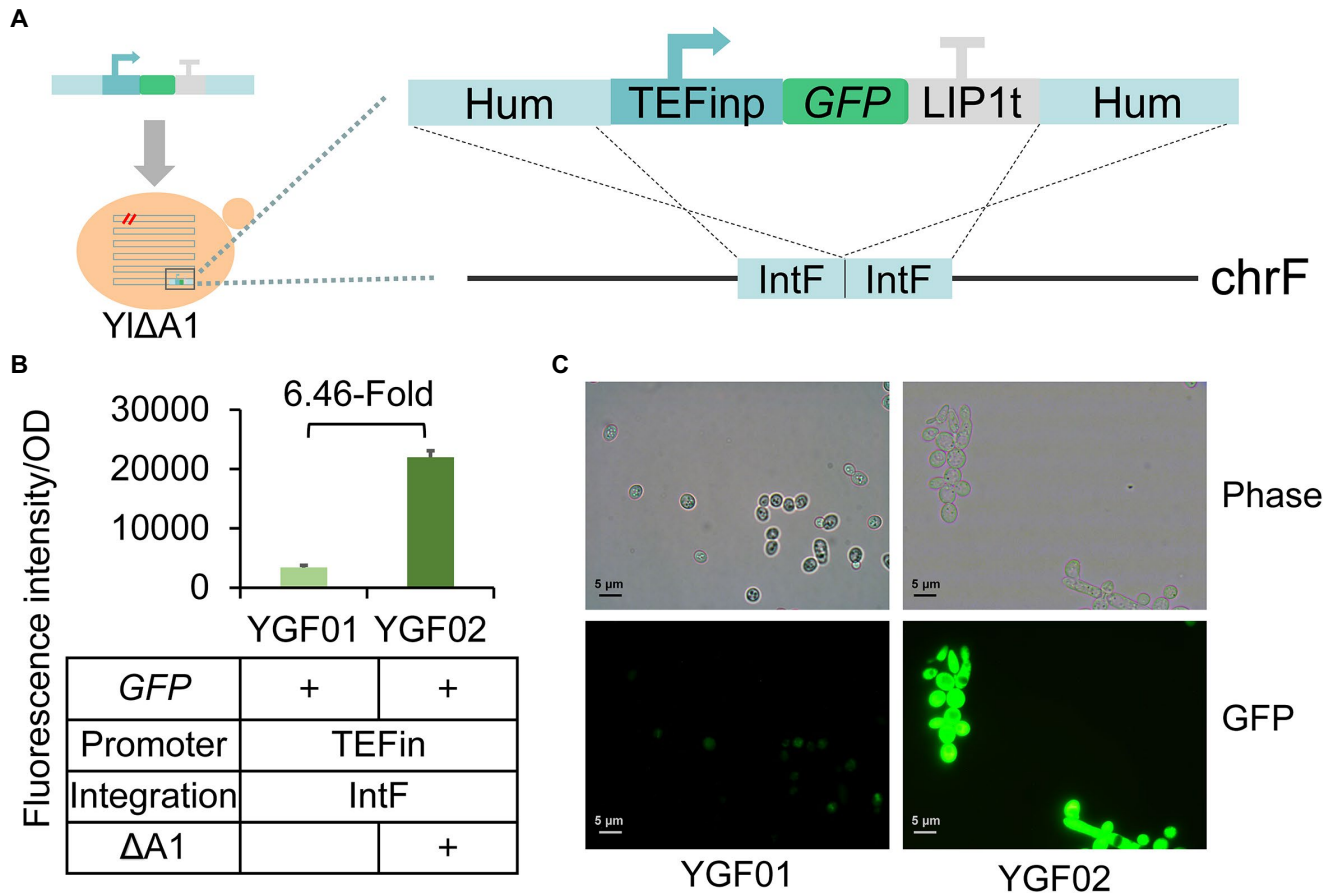
Verification of improved protein expression by *sfGFP* gene expression. **(A)** Schematic diagram of the *sfGFP* insertion cassette used to transform *Y. lipolytica*. The *sfGFP* gene was driven by the TEFin promoter and stopped by the LIP1 terminator, flanking by two 500 bp homologous arms to IntF site. **(B)** The A1 gene disruption increased protein expression. Characterization of protein expression by green fluorescence intensity. All strains were cultured in SC-Leu medium. Values are the mean of three biological replicates ± standard deviation (*n* = 3) after 48 h. **(C)** Fluorescence images of the strains YGF01 and YGF02. The images of *sfGFP* fluorescence were observed by a fluorescence microscope (Olympus CX41, Japan).

To further confirm the increased protein synthesis suggested by transcriptome analysis, we characterized protein expression before and after A1 gene disruption using superfolder green fluorescent protein (*sfGFP*; Pedelacq et al., 2006) as a reporter, which was work well in *Y. lipolytica* (Zhang et al., 2018; Bai et al., 2021). The *sfGFP* gene expressed by constitutive TEFin promoter in pIntF vector was integrated into the IntF site of the ATCC 201249 and YlΔA1 strains through HR (Figure 6A), generating strains of YGF01 to YGF02 (Figure 6B). Compared with the fluorescence intensity of the strain YGF01, the fluorescence intensity of the strain YGF02 that disrupts the A1 gene was increased by about 6.46 times. The fluorescence intensity of the cells was observed by fluorescence microscope, as shown in Figure 6C, the cell growth state of strain YGF02 was no different from that of strain YGF01, but the fluorescence intensity was significantly enhanced, which was consistent with the Figure 6B. These results indicated that the disruption of A1 gene truly increased protein expression, which further corroborates the transcriptome results. And the protein content of WT-FAR and YlΔA1-FAR strains was measured at 48 h, the results did support the above conclusion (Supplementary Table 5).

**Figure 7:**
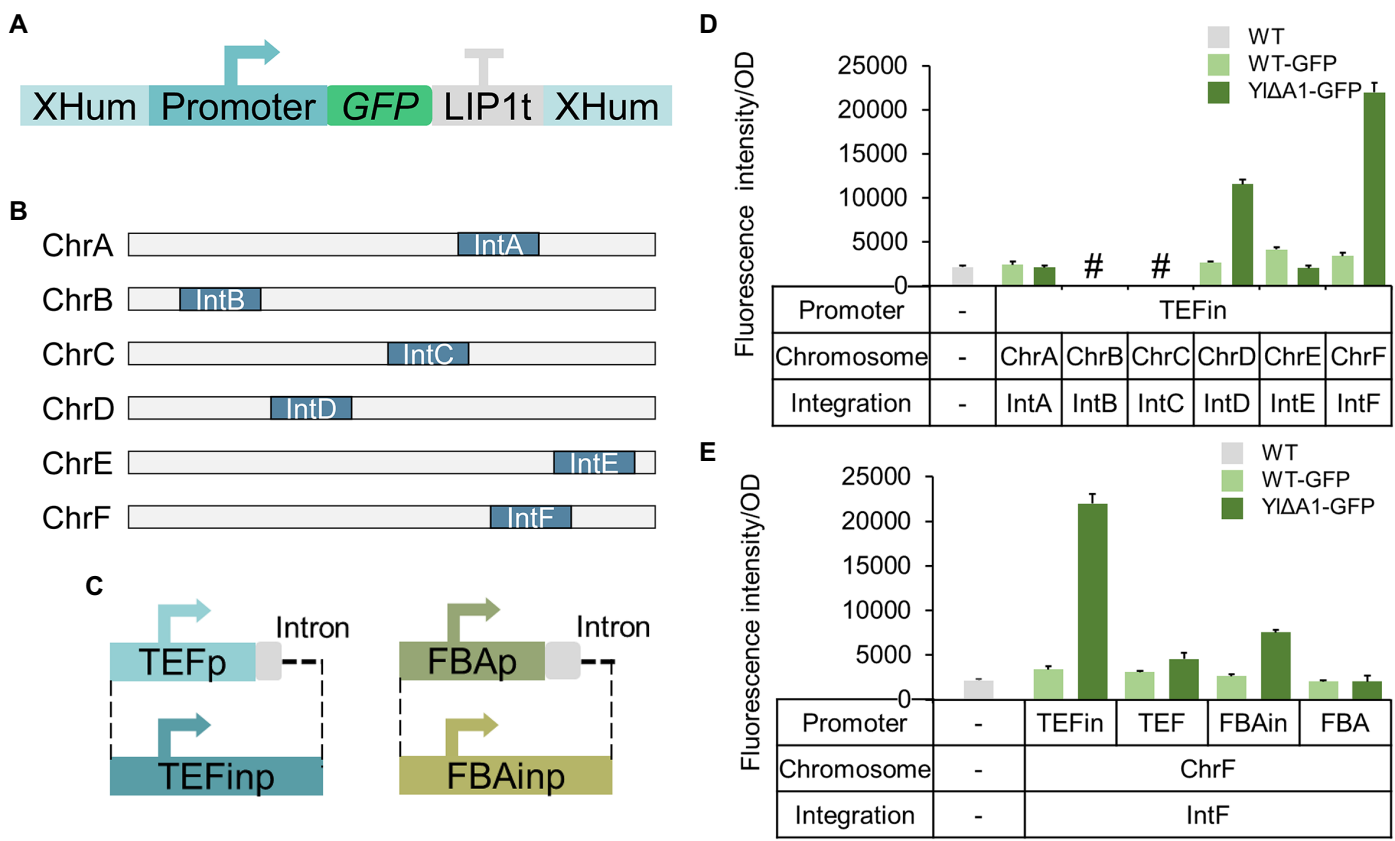
Additional regularities for enhanced expression of exogenous protein. **(A)** Schematic diagram of transformed fragments with different promoters and homologous arms. **(B)** Schematic diagram of the distribution of IntX integration sites on the chromosome in *Y. lipolytica*. **(C)** Schematic diagram of the promoters with or without intron sequences. **(D)** The impact of different integration sites on protein expression from A1 disruption. Characterization of protein expression by green fluorescence intensity. The octothorpe (#) indicated the integration failure of *sfGFP* gene on chromosome B and C. **(E)** The impact of different promoters on protein expression from A1 disruption. Characterization of protein expression by green fluorescence intensity. All strains were cultured in SC-Leu medium. Values are the mean of three biological replicates standard deviation (*n* = 3) after 48 h.

To investigate the regularity of enhanced exogenous protein expression, we next verified whether other promoters and integration sites (Figure 7A) were applicable to the increased protein expression in YlΔA1 strain. Firstly, we selected the IntX location (IntA to IntF; Matthaus et al., 2014) as the integration site, which was commonly used in *Y. lipolytica*. The *sfGFP* gene was integrated into the IntX location of strains ATCC 201249 and YlΔA1, the integration location of the resulting strains is shown in Figure 7B. After 48 h of fermentation, the results were shown in Figure 7D, when the *sfGFP* gene is integrated into chromosomes D and F in the YlΔA1 strain, the green fluorescence intensity was 4.30 and 6.46 times higher than ATCC201249, respectively. But the improvement was not observed when the *sfGFP* gene was integrated into the A and E chromosomes and the integration on the B and C chromosomes failed. Then, we selected two sets of promoters with or without intron sequences (Figure 7C) including TEFin and TEF (Tai and Stephanopoulos, 2013), FBAin and FBA (Hong et al., 2012) to express *sfGFP* gene in strains ATCC 201249 and YlΔA1. The fluorescence intensity of the resulting strains was shown in Figure 7E, the expression of *sfGFP* gene driven by the promoter with intron sequences in YlΔA1 strain was higher than ATCC 201249, but the improvement was not observed when the promoters without intron sequences. This demonstrated that disruption of the A1 gene prefers to enhance gene expression by promoters with intron sequences. And in the above results the Hp4d promoter also contained intron sequence which further proved this conclusion (Figure 1B). Overall, disruption of the A1 gene increased protein expression, which boosted cell viability and may be responsible for the improved cellular synthesis. However, the enhancement of exogenous protein expression from A1 disruption was applicable to D and F chromosomes and promoters with introns.

## Discussion

The regulatory mechanisms of the metabolic networks are extremely complex (Nielsen and Keasling, 2016; Chen et al., 2018; Zhu et al., 2020). Therefore, a substantial set of beneficial gene targets across the genome targets that can facilitate the performance of the strains, in addition to synthetic pathways of a desired product. Multiplex automated genome engineering (MAGE; Wang et al., 2009), trackable multiplex recombineering (TRMR; Warner et al., 2010) and other similar approaches can generate genome-scale mutagenesis for the identification of new targets. However, these techniques have not yet been extensively applied to other non-model organisms. Many non-conventional yeasts and fungi display a high preference for DSBs repair by NHEJ, which is several orders of magnitude than homologous recombination (HR; Wagner and Alper, 2016). And DSBs are widely distributed throughout the genome (Dellino et al., 2019). Therefore, NHEJ-mediated integration confers the randomness and genome-scale unbiased coverage of gene mutations (Liu et al., 2022), which can efficiently generate random mutagenesis across the genome in different microorganisms. NHEJ-mediated integration sometimes can still be produced even when HR is performed to integrate genes in NHEJ-preferred strains, generating unexpected superiors phenotypes with additional insertions (Gao et al., 2014). That requires researchers to value and further investigate incidental phenotypic differences to identify genotypic alterations instead of ignoring them.

In conclusion, we identified a novel target YALI0_A00913g (“A1 gene”) that was disrupted by NHEJ-mediated insertional mutagenesis, through whole-genome sequencing an unexpectedly high fatty alcohol-producing strain obtained incidentally. Transcriptome analysis revealed that disruption of the A1 gene increased protein synthesis, which enhanced cell viability and improved product synthesis. Our research confirmed the importance of potential target excavation in the whole genome and provided a new engineering approach, which is of significant value to further stimulate the biosynthetic potential of for microorganisms.

## Data Availability Statement

The datasets presented in this study can be found in online repositories. The names of the repository/repositories and accession number(s) can be found at: https://www.ncbi.nlm.nih.gov/bioproject/, PRJNA818142; https://www.ncbi.nlm.nih.gov/geo/, GSE199895.

## Author Contributions

YC and HS supervised the study. ML and JZ conceived and designed the experiments. ML and QB performed the experiments. ML, JZ, LF, and QB analyzed the data. YC and ML revised the manuscript. All authors contributed to the article and approved the submitted version.

## Funding

This work was supported by the National Key Research and Development Program of China (2021YFC2104400), the National Natural Science Foundation of China (NSFC 21621004 and NSFC 22078240), the Natural Science Foundation of Tianjin City (19JCQNJC09200), and the Young Elite Scientists Sponsorship Program by Tianjin (TJSQNTJ-2018-16).

## Conflict of Interest

The authors declare that the research was conducted in the absence of any commercial or financial relationships that could be construed as a potential conflict of interest.

## Publisher’s Note

All claims expressed in this article are solely those of the authors and do not necessarily represent those of their affiliated organizations, or those of the publisher, the editors and the reviewers. Any product that may be evaluated in this article, or claim that may be made by its manufacturer, is not guaranteed or endorsed by the publisher.

## Footnotes

^1^ http://soap.genomics.org.cn/soapaligner.html

^2^ http://soap.genomics.org.cn/soapsnp.html

^3^ https://report.bgi.com
