# Supplementary material for: Non-homologous End Joining-Mediated Insertional Mutagenesis Reveals a Novel Target for Enhancing Fatty Alcohols Production in Yarrowia lipolytica
Source: Front Microbiol. 2022 Apr 25;13:898884. doi: 10.3389/fmicb.2022.898884 (PMC9082995; doi:10.3389/fmicb.2022.898884)
Supplement: Supplementary file 1 [file Data_Sheet_1.docx]

Supplementary materials

1. **Supplementary Data**

**Supplementary Data 1. Codon optimized gene sequences involved in this study.**

> *FAR* from *Marinobacter aquaeolei* VT8

ATGGCCATCCAGCAGGTGCACCACGCTGACACCTCTTCCTCTAAGGTCCTGGGTCAGCTGCGAGGCAAGCGAGTGCTGATTACCGGCACCACCGGATTCCTGGGCAAGGTGGTCCTGGAGCGACTGATCCGAGCCGTCCCCGACATCGGCGCTATCTACCTGCTGATTCGAGGAAACAAGCGACACCCCGACGCCCGATCCCGATTCCTGGAGGAGATCGCTACCTCCTCTGTGTTCGACCGACTGCGAGAGGCCGACTCTGAGGGTTTCGACGCTTTCCTGGAGGAGCGAATCCACTGTGTCACCGGAGAGGTGACCGAGGCTGGTTTCGGAATTGGTCAGGAGGACTACCGAAAGCTGGCCACCGAGCTGGACGCTGTCATTAACTCCGCCGCTTCTGTGAACTTCCGAGAGGAGCTGGACAAGGCCCTGGCTATCAACACCCTGTGTCTGCGAAACATTGCCGGAATGGTCGACCTGAACCCCAAGCTGGCTGTGCTGCAGGTGTCTACCTGCTACGTGAACGGAATGAACTCCGGTCAGGTCACCGAGTCTGTGATCAAGCCTGCTGGAGAGGCTGTCCCTCGATCTCCCGACGGTTTCTACGAGATCGAGGAGCTGGTCCGACTGCTGCAGGACAAGATTGAGGACGTGCAGGCCCGATACTCCGGCAAGGTCCTGGAGCGAAAGCTGGTGGACCTGGGTATTCGAGAGGCTAACCGATACGGCTGGTCTGACACCTACACCTTCACCAAGTGGCTGGGCGAGCAGCTGCTGATGAAGGCCCTGAACGGACGAACCCTGACCATCCTGCGACCCTCCATCATTGAGTCTGCTCTGGAGGAGCCTGCTCCT

GGTTGGATTGAGGGAGTGAAGGTCGCCGACGCTATCATTCTGGCCTACGCTCGAGAGAAGGTGACCCTGTTCCCCGGCAAGCGATCCGGCATCATTGACGTGATCCCCGTCGACCTGGTGGCCAACTCCATCATTCTGTCTCTGGCTGAGGCTCTGGGAGAGCCTGGACGACGACGAATCTACCAGTGTTGCTCCGGCGGAGGTAACCCCATCTCTCTGGGAGAGTTCATTGACCACCTGATGGCCGAGTCCAAGGCTAACTACGCCGCTTACGACCACCTGTTCTACCGACAGCCCTCTAAGCCCTTCCTGGCCGTCAACCGAGCTCTGTTCGACCTGGTCATCTCCGGAGTGCGACTGCCCCTGTCTCTGACCGACCGAGTGCTGAAGCTGCTGGGTAACTCCCGAGATCTGAAGATGCTGCGAAACCTGGACACCACCCAGTCTCTGGCCACCATCTTCGGATTCTACACCGCTCCCGACTACATTTTCCGAAACGACGAGCTGATGGCCCTGGCTAACCGAATGGGAGAGGTCGACAAGGGTCTGTTCCCCGTGGACGCCCGACTGATCGACTGGGAGCTGTACCTGCGAAAGATTCACCTGGCCGGCCTGAACCGATACGCTCTGAAGGAGCGAAAGGTGTACTCTCTGAAGACCGCCCGACAGCGAAAGAAGGCCGCTTAA

> *sfGFP*

ATGCGAAAGGGTGAGGAGCTGTTCACCGGTGTGGTGCCCATCCTGGTGGAGCTGGACGGCGACGTCAACGGTCACAAGTTCTCTGTGCGAGGTGAGGGCGAGGGCGACGCCACTAACGGTAAACTGACCCTGAAGTTCATTTGTACCACCGGTAAACTGCCCGTGCCCTGGCCCACCCTGGTCACTACCCTGACCTACGGCGTGCAGTGTTTCGCCCGATACCCCGACCACATGAAGCAGCACGACTTCTTCAAGTCCGCCATGCCCGAGGGCTACGTGCAGGAGCGAACCATCTCCTTCAAGGACGACGGCACCTACAAGACCCGAGCCGAGGTGAAGTTCGAGGGTGACACCCTGGTGAACCGAATCGAGCTGAAGGGTATCGACTTCAAGGAGGACGGTAACATCCTGGGCCACAAGCTGGAGTACAACTTCAACTCTCACAACGTCTACATCACCGCCGACAAGCAGAAGAACGGCATCAAGGCCAACTTCAAGATCCGACACAACGTCGAGGACGGCTCCGTGCAGCTGGCCGACCACTACCAGCAGAACACCCCCATCGGTGACGGTCCCGTGCTGCTGCCCGACAACCACTACCTGTCCACCCAGTCCGTCCTGTCTAAGGACCCCAACGAGAAGCGAGATCATATGGTGCTGCTGGAGTTCGTGACCGCCGCCGGCATCACCCACGGTATGGACGAGCTGTACAAGTAA

1. **Supplementary Figures and Tables**

## Supplementary Figures


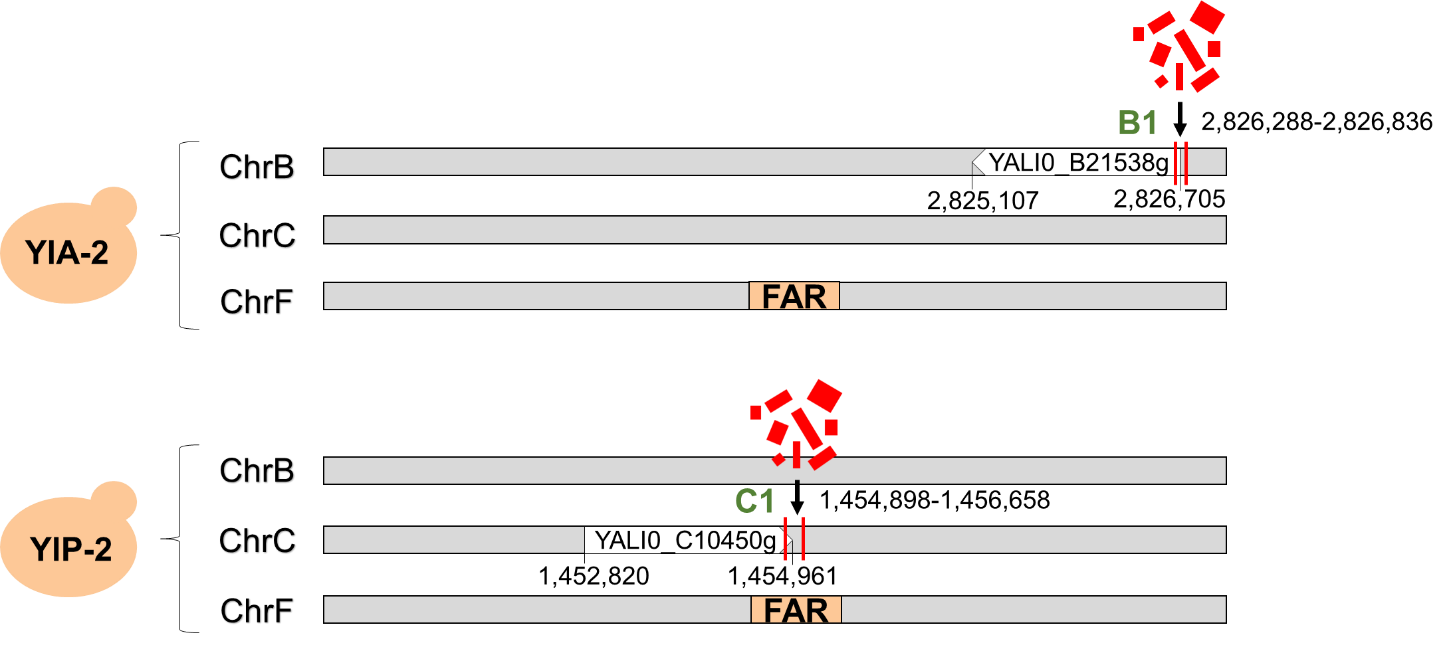


**Supplementary Figure 1. Whole-genome sequencing determined gene disruption in fatty alcohol low-yielding strains.**

YlA-2 and YlP-2 are the low fatty alcohol production strains. The gene disruption caused by the integration of broken vector fragments into chromosomes B and C was marked with red vertical line, named B1 and C1 sites respectively. The B1 site was located in the 2,826,288-2,826,836 region of chromosomes B, and the C1 site was in the 1,454,898-1,456,658 region of chromosomes C.


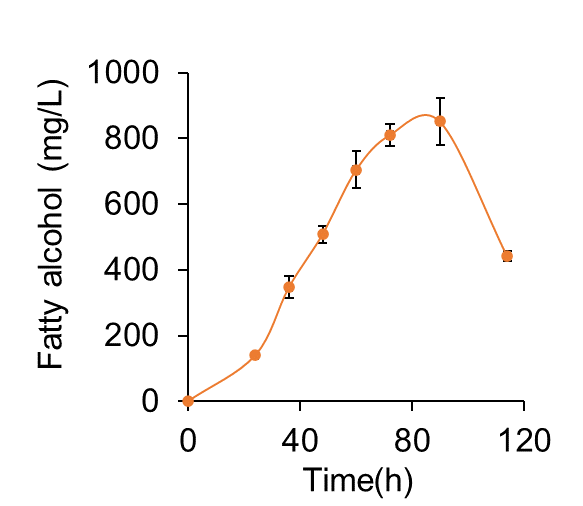


**Supplementary Figure 2. Fatty alcohol production curve of YlA1-FAR strain.**

**Supplementary Figure 3. Amino acid sequence alignment of A1 with YND1.**

**
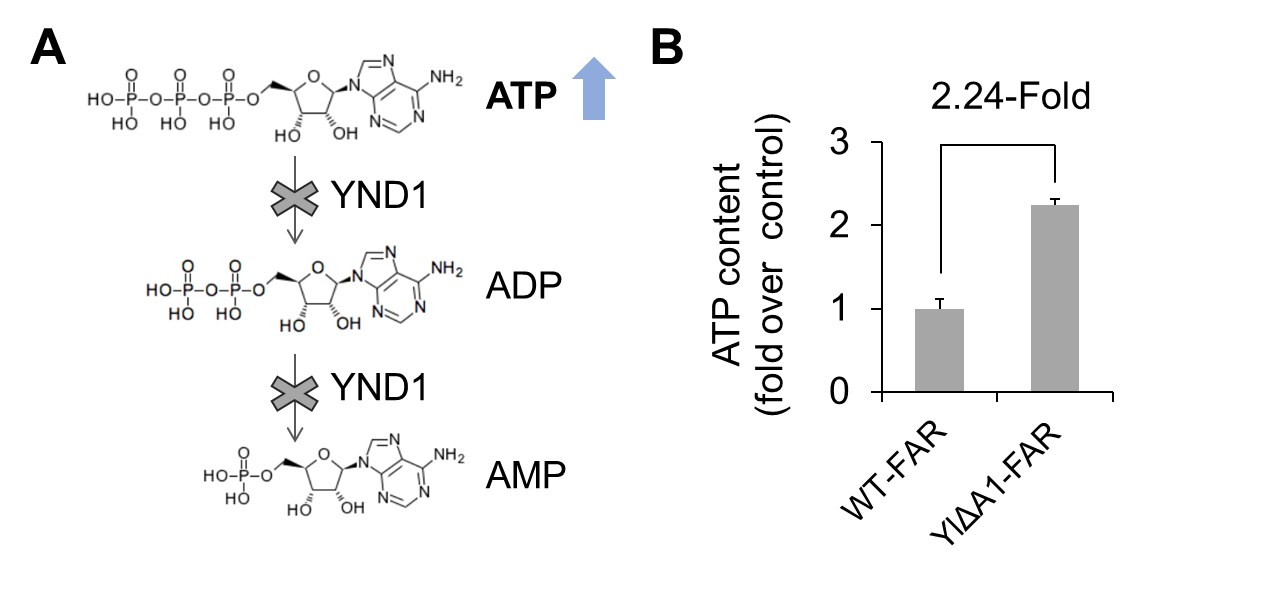
**

**Supplementary Figure 4. Metabolic pathway for the *YND1* gene and changes in ATP content owing to disruption of A1 gene.**

A. The A1 gene is weakly similar to the *YND1* gene of *Saccharomyces cerevisiae*, which encodes Golgi apyrase that generally hydrolyzes both ATP and ADP. Disruption of the *YND1* gene results in ATP accumulation.

B. The ATP level of WT-FAR and Y1ΔA1-FAR strains.


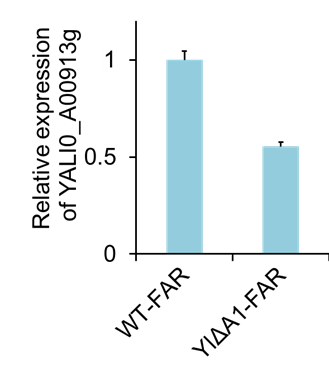


**Supplementary Figure 5. Transcript levels of the YALI0_A00913g (A1) gene before and after disruption.**

Results are presented as mean ± s. d. of three biological replicates.

## Supplementary Tables

**Supplementary Table 1. Strains used in this study.**

| Strains | Relative characteristics | Source |
| --- | --- | --- |
| *Yarrowia lipolytica*  ATCC 201249 | MATA *ura3-302 leu2-270 lys8-11 PEX17-HA* | (Gao et al., 2016) |
| Yl0301 | ATCC 201249 *ΔPEX10* | This study |
| YlA-1 | ATCC 201249 harboring pINA1269-*FAR* | This study |
| YlA-2 | ATCC 201249 harboring pINA1269-*FAR* | This study |
| YlP-1 | Yl0301 harboring pINA1269-*FAR* | This study |
| YlP-2 | Yl0301 harboring pINA1269-*FAR* | This study |
| YlΔA1 | ATCC 201249 Δ*A1* | This study |
| YlΔE1 | ATCC 201249 Δ*E1* | This study |
| WT-FAR | ATCC 201249 IntF:: pTEFin-*FAR*-lip1t, LEU2 | This study |
| YlΔA1-FAR | YlΔ*A1* IntF:: pTEFin-*FAR*-lip1t, LEU2 | This study |
| YlΔE1-FAR | YlΔ*E1* IntF:: pTEFin-*FAR*-lip1t, LEU2 | This study |
| YGF01 | ATCC 201249 IntF:: pTEFin-*GFP*-lip1t, LEU2 | This study |
| YGF02 | YlΔ*A1* IntF:: pTEFin-*GFP*-lip1t, LEU2 | This study |
| YGF03 | ATCC 201249 IntA:: pTEFin-*GFP*-lip1t, LEU2 | This study |
| YGF04 | YlΔ*A1* IntA:: pTEFin-*GFP*-lip1t, LEU2 | This study |
| YGF05 | ATCC 201249 IntD:: pTEFin-*GFP*-lip1t, LEU2 | This study |
| YGF06 | YlΔ*A1* IntD:: pTEFin-*GFP*-lip1t, LEU2 | This study |
| YGF07 | ATCC 201249 IntE:: pTEFin-*GFP*-lip1t, LEU2 | This study |
| YGF08 | YlΔ*A1* IntE:: pTEFin-*GFP*-lip1t, LEU2 | This study |
| YGF09 | ATCC 201249 IntF:: pTEF-*GFP*-lip1t, LEU2 | This study |
| YGF10 | YlΔ*A1* IntF:: pTEF-*GFP*-lip1t, LEU2 | This study |
| YGF11 | ATCC 201249 IntF:: pFBAin-*GFP*-OCTt, LEU2 | This study |
| YGF12 | YlΔ*A1* IntF:: pFBAin-*GFP*-OCTt, LEU2 | This study |
| YGF13 | ATCC 201249 IntF:: pFBA-*GFP*-OCTt, LEU2 | This study |
| YGF14 | YlΔ*A1* IntF:: pFBA-*GFP*-OCTt, LEU2 | This study |

**Supplementary Table 2. Plasmids used in this study.**

| Plasmids | Relative characteristics | Source |
| --- | --- | --- |
| pINA 1269 | *Y. lipolytica* integrative plasmid, hp4d promoter, XPR2 terminator, LEU2 selection marker, AmpR | (Madzak et al., 2000; Nicaud et al., 2002; Xuan et al., 1990) |
| pIntF | IntFup-PTEFin-lip1t-leu-IntFdn cassette in PUC57 | This study |
| pIntF-TEF | IntFup-PTEF-lip1t-leu-IntFdn cassette in PUC57 | This study |
| pIntF-FBAin | IntFup-PFBAin-lip1t-leu-IntFdn cassette in PUC57 | This study |
| pIntF-FBA | IntFup-PFBA-lip1t-leu-IntFdn cassette in PUC57 | This study |
| pIntE | IntEup-PTEFin-lip1t-leu-IntEdn cassette in PUC57 | This study |
| pIntA | IntAup-PTEFin-lip1t-leu-IntAdn cassette in PUC57 | This study |
| pIntB | IntBup-PTEFin-lip1t-leu-IntBdn cassette in PUC57 | This study |
| pIntC | IntCup-PTEFin-lip1t-leu-IntCdn cassette in PUC57 | This study |
| pIntD | IntDup-PTEFin-lip1t-leu-IntDdn cassette in PUC57 | This study |
| pINA1269-*FAR* | Codon-optimizes FAR gene was cloned into the BamHI/KpnI site of pINA1269 | This study |
| pIntF-*FAR* | *FAR cassette in pIntF* | This study |
| pIntF-*GFP* | *GFP* cassette in pIntF | This study |
| pIntF-TEF-*GFP* | *GFP* cassette in pIntF-TEF-*FAR* | This study |
| pIntF-FBAin-*GFP* | *GFP* cassette in pIntF-FBAin-*FAR* | This study |
| pIntF-FBA-*GFP* | *GFP* cassette in pIntF-FBA-*FAR* | This study |
| pIntE-*GFP* | *GFP* cassette in pIntE-*GFP* | This study |
| pIntA-*GFP* | *GFP* cassette in pIntA-*GFP* | This study |
| pIntB-*GFP* | *GFP* cassette in pIntB-*GFP* | This study |
| pIntC-*GFP* | *GFP* cassette in pIntC-*GFP* | This study |
| pIntD-*GFP* | *GFP* cassette in pIntD-*GFP* | This study |
| PMCS-URA | URA3 marker and guide RNA expression cassette in pMCSCen1 | (Zhang et al., 2019) |
| CRI-PART2 | Cas9 expression cassette in PUC57 | (Zhang et al., 2019) |
| PMCS-*DGA1* | Cas9 expression cassette in pMCSCen1 with *DGA1* gRNA | This study |
| PMCS-*PEX10* | Cas9 expression cassette in pMCSCen1 with *PEX10* gRNA | This study |
| PMCS-*A1* | Cas9 expression cassette in pMCSCen1 with *A1* gRNA | This study |
| PMCS-*E1* | Cas9 expression cassette in pMCSCen1 with *E1* gRNA | This study |

**Supplementary Table 3. Primer sequences used in this work.**

| Primer | Sequence | Source |
| --- | --- | --- |
| AUp-F | ACCATGATTACGCCAAGCTTCCACAGGCTCTTCAAGTCACCA | This work |
| AUp-R | CCGCCAACCCGGTCTCTCAAGACCAAGACTGTTCCCAAGGC | This work |
| ADown-F | AGCTTGTTACTGTATATTCGTCTGGACTCGGCCTTCGC | This work |
| ADown-R | AGCTCGGTACCCGGGGATCCGCTGGTTGACTTTGACTCCAACTG | This work |
| BUp-F | ACCATGATTACGCCAAGCTTCCCACAGTTCTCACTCAGATCATGG | This work |
| BUp-R | CCGCCAACCCGGTCTCTCACGAGAGCAGTAAATACTGCCAG | This work |
| BDown-F | AGCTTGTTACTGTATATTCGACCTGCTCCTGCACCTAAGT | This work |
| BDown-R | AGCTCGGTACCCGGGGATCCCATAAGACGCCTCGTTGCTC | This work |
| CUp-F | ACCATGATTACGCCAAGCTTCTTCTGCTCGACGAGATAGGC | This work |
| CUp-R | CCGCCAACCCGGTCTCTCAAGTGACCAAACAGCCGA | This work |
| CDown-F | AGCTTGTTACTGTATATTCGCAACGGGTATGCACTTTGTGAC | This work |
| CDown-R | AGCTCGGTACCCGGGGATCCTCGCTTGCCTTCGCTTAGGAC | This work |
| DUp-F | ACCATGATTACGCCAAGCTTTGTGAAGACGGCCAGATACAGAC | This work |
| DUp-R | CCGCCAACCCGGTCTCTAATGTCTGTACCTGACGTTATCTCTAT | This work |
| DDown-F | AGCTTGTTACTGTATATTCGGACATGATACATTCTGTTGCTGGA | This work |
| DDown-R | AGCTCGGTACCCGGGGATCCACCGTAAAGACGGGAACCAC | This work |
| EUp-F | ACCATGATTACGCCAAGCTTTGAGAGCAAGCGATACACATGT | This work |
| EUp-R | CCGCCAACCCGGTCTCTGTATATTATACCTCACACAGCGGTCC | This work |
| EDown-F | AGCTTGTTACTGTATATTCGGCTTCGAGTTTAAGCTGATTAAACC | This work |
| EDown-R | AGCTCGGTACCCGGGGATCCTGTTTTAGCTTGAGCCGTTTTC | This work |
| TEF-F | CGGCAAAACCACCTGTAAAGAGACCGGGTTGGCG | This work |
| TEF-R | TTTAGGAGACGCGTCTCCCATTTTGAATGATTCTTATACTCAGAAG | This work |
| FBAin-F | CGGCAAAACCACCTGTAAACAGTGTACGCAGTACTATAGAGGAA | This work |
| FBAin-r | TTTAGGAGACGCGTCTCCGTTAGTTTGTGTAGAGAGTGTGTGT | This work |
| FBA-F | CGGCAAAACCACCTGTAAACAGTGTACGCAGTACTATAGAGGA | This work |
| FBA-R | TTTAGGAGACGCGTCTCCCATTGTGTGATGTGTAGTTTAGATTT | This work |
| BsmbI-IntF-*FAR*-F | CGTCTCCGCAGGCCATCCAGCAGGTGCAC | This work |
| BsmbI-IntF-*FAR*-R | CGTCTCCTTTAAGCGGCCTTCTTTCGC | This work |
| BsmbI-IntF-*GFP*-F | CGTCTCCGCAGCGAAAGGGTGAGGAGCTGTTCAC | This work |
| BsmbI-IntF-*GFP*-R | CGTCTCCTTTACTTGTACAGCTCGTCCATACCGTG | This work |
| PEX10-cas9-F | ACGTGCCCAGCCCGGAAACATGGA | This work |
| PEX10-cas9-R | AAACTCCATGTTTCCGGGCTGGGC | This work |
| A1-cas9-F | ACGTCGGTGGCCAAGAGGACGTCA | This work |
| A1-cas9-R | AAACTGACGTCCTCTTGGCCACCG | This work |
| E1-cas9-F | ACGTATGCTCCTACTCCTCTTCAC | This work |
| E1-cas9-R | AAACGTGAAGAGGAGTAGGAGCAT | This work |

**Supplementary Table 4. Insertion.**

| Strain | Parent | Insertion site | Disrupted Gene |
| --- | --- | --- | --- |
| YlA-1 | ATCC 201249 | Chr. A: 118335-118543 | YALI0_A00913g |
| YlA-2 | ATCC 201249 | Chr. B: 2826288-2826836 | YALI0_B21538g |
| YlP-1 | Yl0301 | Chr. E: 4134688-4134892 | YALI0_E34687g |
| YlP-2 | Yl0301 | Chr. C: 1454898-1456658 | YALI0_C10450g |

**Supplementary Table 5. Protein content of WT-FAR far and YlΔA1-FAR.**

| Strain | Protein concentration (μg/μl) | Total protein (μg) |
| --- | --- | --- |
| WT-FAR | 1.35 | 646.70 |
| YlΔA1-FAR | 2.18 | 1046.88 |

Gao, S., Tong, Y., Wen, Z., Zhu, L., Ge, M., Chen, D., Jiang, Y., Yang, S., 2016. Multiplex gene editing of the *Yarrowia lipolytica* genome using the CRISPR-Cas9 system. J Ind Microbiol Biotechnol. 43**,** 1085-93.

Madzak, C., Treton, B., Blanchin-Roland, S., 2000. Strong hybrid promoters and integrative expression/secretion vectors for quasi-constitutive expression of heterologous proteins in the yeast *Yarrowia lipolytica*. J Mol Microbiol Biotechnol. 2**,** 207-16.

Nicaud, J. M., Madzak, C., van den Broek, P., Gysler, C., Duboc, P., Niederberger, P., Gaillardin, C., 2002. Protein expression and secretion in the yeast *Yarrowia lipolytica*. FEMS Yeast Res. 2**,** 371-9.

Xuan, J. W., Fournier, P., Declerck, N., Chasles, M., Gaillardin, C., 1990. Overlapping reading frames at the LYS5 locus in the yeast *Yarrowia lipolytica*. Molecular and Cellular Biology. 10**,** 4795-4806.

Zhang, J.-L., Cao, Y.-X., Peng, Y.-Z., Jin, C.-C., Bai, Q.-Y., Zhang, R.-S., Liu, D., Yuan, Y.-J., 2019. High production of fatty alcohols in *Yarrowia lipolytica* by coordination with glycolysis. Science China Chemistry. 62**,** 1007-1016.
